# The Network of Tumor Microtubes: An Improperly Reactivated Neural Cell Network With Stemness Feature for Resistance and Recurrence in Gliomas

**DOI:** 10.3389/fonc.2022.921975

**Published:** 2022-06-29

**Authors:** Xinyue Wang, Jianhao Liang, Haitao Sun

**Affiliations:** ^1^ Clinical Biobank Center, Microbiome Medicine Center, Department of Laboratory Medicine, Zhujiang Hospital, Southern Medical University, Guangzhou, China; ^2^ Neurosurgery Center, The National Key Clinical Specialty, The Engineering Technology Research Center of Education Ministry of China on Diagnosis and Treatment of Cerebrovascular Disease, Guangdong Provincial Key Laboratory on Brain Function Repair and Regeneration, The Neurosurgery Institute of Guangdong Province, Zhujiang Hospital, Southern Medical University, Guangzhou, China; ^3^ Key Laboratory of Mental Health of the Ministry of Education, Guangdong–Hong Kong–Macao Greater Bay Area Center for Brain Science and Brain–Inspired Intelligence, Southern Medical University, Guangzhou, China

**Keywords:** glioma, tumor microtubes, tunneling nanotubes, cancer stem cells, resistance, heterogeneity, invasion, brain tumor microenvironment

## Abstract

Gliomas are known as an incurable brain tumor for the poor prognosis and robust recurrence. In recent years, a cellular subpopulation with tumor microtubes (TMs) was identified in brain tumors, which may provide a new angle to explain the invasion, resistance, recurrence, and heterogeneity of gliomas. Recently, it was demonstrated that the cell subpopulation also expresses neural stem cell markers and shares a lot of features with both immature neurons and cancer stem cells and may be seen as an improperly reactivated neural cell network with a stemness feature at later time points of life. TMs may also provide a new angle to understand the resistance and recurrence mechanisms of glioma stem cells. In this review, we innovatively focus on the common features between TMs and sprouting axons in morphology, formation, and function. Additionally, we summarized the recent progress in the resistance and recurrence mechanisms of gliomas with TMs and explained the incurability and heterogeneity in gliomas with TMs. Moreover, we discussed the recently discovered overlap between cancer stem cells and TM-positive glioma cells, which may contribute to the understanding of resistant glioma cell subpopulation and the exploration of the new potential therapeutic target for gliomas.

## 1 Introduction

Gliomas represented approximately 25% of primary CNS (central nervous system) tumors and 80% of malignant tumors (1), characterized by poor prognosis with a median overall survival of approximately 15 months (2). Gliomas share a lot of morphological features with glial cells in the normal brain, such as astrocytes, oligodendrocytes, and ependymal cells, consequently classified morphologically (3). Of note, there is an increasing recognition of the important role of molecular characteristics in the classification of gliomas, such as the mutation of Isocitrate dehydrogenase (IDH) and 1p/19q co-deletion, for the distinctive clinical manifestations of heterogeneous cell subpopulations (3). Gliomas are still known as an incurable brain tumor for the poor prognosis and robust recurrence (3). Increasing attention has been paid to the mechanism related to their widespread infiltration and robust resistance and self-repairment, particularly the heterogeneity of the resistant cellular subpopulation (4). For example, a cellular subpopulation of glioma cells with NSC (neural stem cell) marker expression, usually forming tumor microtubes (TMs), was identified (5–7), which may be related to the high resistance of gliomas to all existing standard therapies and recurrence (8–11). Furthermore, the TM-positive cell subpopulation may provide a new angle on the explanation of the mechanisms and the heterogeneity of glioma cells (12).

Tumor microtubes are ultralong membrane protrusions extended by glioma cells, which are demonstrated to facilitate glioma cell widespread infiltration and treatment resistance (12, 13). Two morphologically, molecularly, and functionally different TM subtypes have been identified: non-connecting ones, which are crucial for glioma invasion and proliferation, and interconnecting ones, which build Cx43-separated membrane tube connections between individual glioma cells (14). Ramírez-Weber and his colleagues first detected membrane tubes for intercellular connections in *Drosophila* development (15). Subsequently, accumulating cell types have been identified to form and utilize membrane tubes to exchange various cellular components, which are exemplified by organelles (16), pathogens including HIV (17–19) and prions (20), and genetic material (12, 21). The membrane tubes have gained various names: membrane nanotubes, tunneling nanotubes, and cytonemes (12). For the distinctive characteristics of the membrane tubes in gliomas, they were specially termed “tumor microtubes” (TMs) (12).

## 2 TMs and TM-Positive Cells Share a Lot of Features With Sprouting Axons and Immature Neurons

Recently, it was demonstrated that the dysregulated and disordered glioma progression and malignancy are, in essence, parallel to the directed and ordered CNS development and function based on the morphological characteristics, mechanism of cellular proliferation, migration, and communication (22, 23). In line with this, TMs and the TM-positive glioma cell network share a lot of features with sprouting axons and immature neuroblasts (**Table 1**).

**Table 1:** Characteristics of TMs in comparison with human sprouting neuron axons.

Feature	TMs	Axons
**Width**	Mean, 1.7 μm	0.08–0.4 μm Maximum, 20 μm
**Length**	Maximum,>500 μm	Minimum,<1 mm Maximum,>1 m
**Lifetime**	Days, up to 200	years
**Content**		
**Actin**	+	+
**Mitochondria**	+	+
**Protein**	+	+
**Endoplasmic reticulum**	+	+
**Microvesicles**	+	+
**Microtubules**	+	+
**Myosin X**	–	+
**Myosin IIa**	+	–
**β III tubulin**	–	+
**Voltage-gated Ca^2+^ channel**	+	+
**Ttyh1**	+	+
**Gap43**	+	+
**Cx43**	+	+
**Functions**		
**Nucleus transmission**	+	?
**cell migration**	+	?
**Mitochondria transmission**	+	+
**Protein transmission**	+	+
**Propagation of ICWs**	+	+
**Pathogen spread**	+	+
**Microvesicle transmission**	+	+

+: positive; -: negative; ?: uncertain.

### 2.1 Morphology and Formation

**Figure 1:**
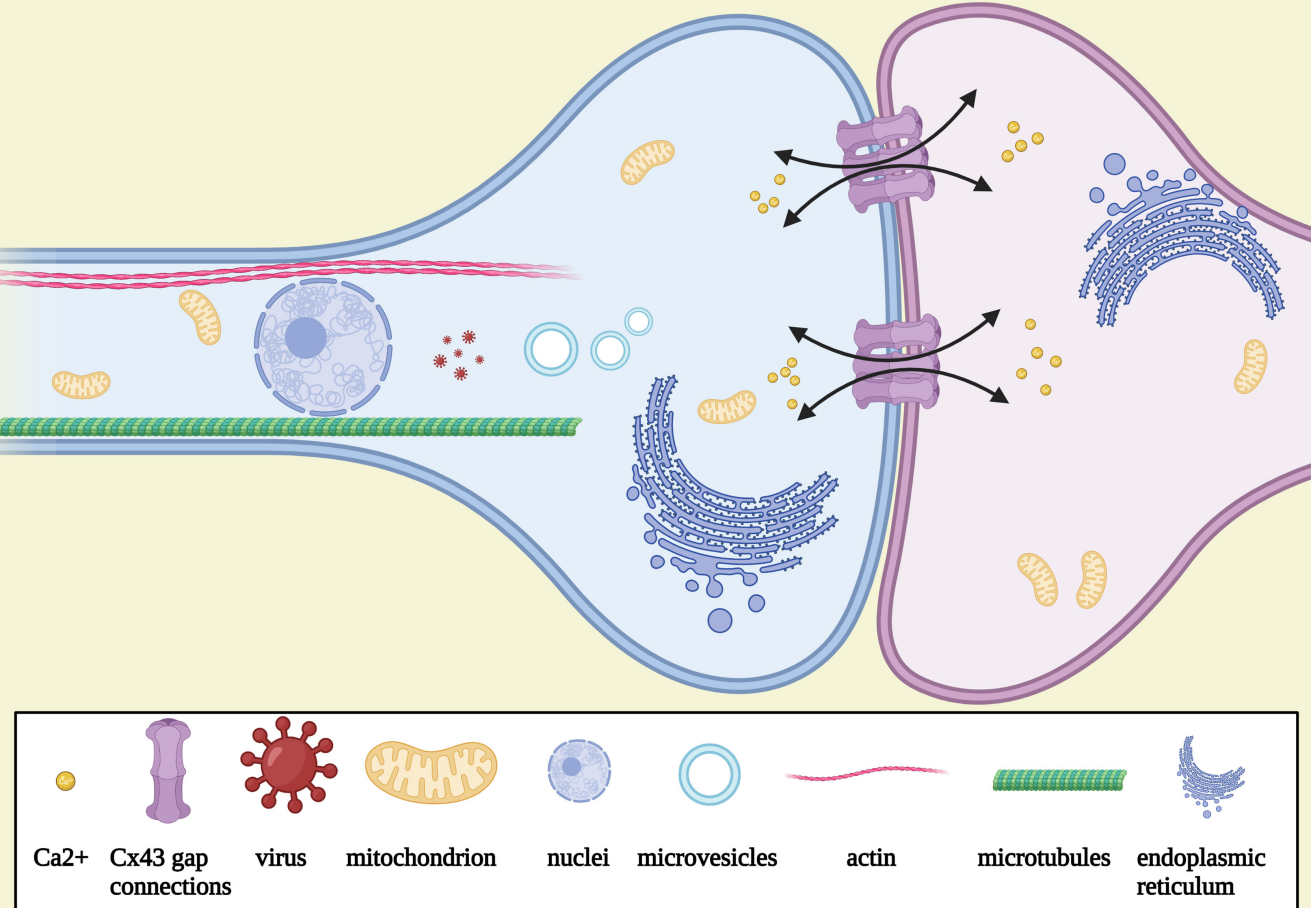
Schematic illustration of the morphology and the function of TMs. Tumor microtube is a continuation of the membrane of a glioma cell and extends to another cell separated with gap junctions connexin 43, which is rich of myosin IIa, actins, microtubules, mitochondria, microvesicles, and endoplasmic reticulum. Nuclei could be seen to travel in TMs after mitosis. Intracellular Ca2+ exchange *via* Cx43.

Different TM subtypes are morphologically and molecularly heterogeneous. A non-connecting tumor microtube is an ultralong membrane protrusion extended by a glioma cell, while an interconnecting tumor microtube is a continuation of the membrane of a glioma cell and extends to another cell separated with gap junctions (**Figure 1**) (12, 14). TMs are 1.7 μm in width on average, and the maximum length reaches more than 500 μm, while tunneling nanotubes are less than 1 μm in width and 30 μm in length on average; the life span of a tumor microtube reaches more than 200 days, while that of tunneling nanotubes is up to 60 min (**Table 1**) (24). It can also be seen *in vivo* that the leading edges of invasive TMs are morphologically parallel with the axonal growth cones of normal sprouting axons (12, 25). Together, these findings suggest morphological parallels between invasive TMs and normal sprouting axons. Additionally, immunohistochemistry showed that the tumor microtubes were rich of myosin IIa, actins, and microtubules, which play an important role in the generation of contractile forces necessary for the movement of glioma cells (12). Likewise, myosin, actin, and microtubules are also known to be rich in the protrusions of neural precursor cells for migration (26). Furthermore, three key molecular players of TMs have been identified: Ttyh1 (14), Gap43 (growth-associated protein 43) (12), and Connexin 43 (12). The gap junction protein connexin 43 is highly expressed at TMs integrated into the network (12). Cx43 immunoreactivity was most frequently only expressed at one end of a TM connecting two cells, suggesting that the one end of a TM is continuous with the cell membrane; the other end is a membrane boundary separated with a gap junction (12). Since the presence of Cx43 is usually accompanied with poor prognosis while the absence of Cx43 is accompanied with a reduced tumor size and improved survival, it was once viewed as an important molecular driver of tumor microtubes. However, some controversial views suggest that Cx43 did not always promote the growth and function of tumor microtubes (27). Recently, it was demonstrated that Cx43 may play an important role in the communication and maintain the integrity of the network, which is exemplified by empowering gliomas to acquire resistance to oxidative stress (28). Gap43 was demonstrated to be a key molecular player in driving TM outgrowth, which is highly expressed in the growth of cone-like tips of the TMs, driving the growth of TMs and TM-dependent astrocytoma cell migrations (12, 24). The knockdown of GAP-43 in mouse brain blocked both TM-non-connected glioma cell invasion and proliferation and intercellular TM connection (12). According to the recent research in mouse models, Ttyh1 also plays a key role in the growth of invasive non-connecting TMs, while the knockdown of Ttyh1 contributed to a dramatically declined proportion of invasive TM-non-connected glioma cells (14). Of note, TM-connected glioma cells appear to be uncompromised by interference with Ttyh1; it was shown that TM-connected glioma cells were not affected in the absence of Ttyh1 (14). Together, these findings suggest that the two TM subtypes are molecularly and functionally heterogeneous. Similarly, Ttyh1 and Gap43 are also highly concentrated in axonal growth cones during neurite outgrowth, driving developmental and regenerative axon growth (29–31) and neuronal progenitor cell migrations (32). The overexpression of Ttyh1 and Gap43 appears to induce neurite outgrowth not only in neuronal (31, 33) but also in non-neuronal cells (34), while the downregulation of expression of them inhibit the neurite outgrowth (35). Furthermore, a recent study in mouse brain showed that intercellular adhesion and signaling provided by p120-catenin-dependent adherens junctions is crucial for both TM-non-connected glioma cell invasion and the TM-connected network (36), which may be highly reminiscent of epithelial tumors regulated by p120 signaling for anchorage-independent growth, anoikis, resistance, and metastasis (37–39).

### 2.2 Function

#### 2.2.1 Communication Network

Interconnecting TMs are shown to be involved in the molecular, organelle, and vesicle transport and intercellular calcium wave (ICW) propagation (12). Cells can be electrically coupled over long distances *via* membrane tubes associated with gab junctions (12). Thus, interconnecting TMs can mediate the bidirectional spread of ICWs between connecting cells through gap junctions (12). ICWs play an important role in the communication of glioma cells with each other and in the coordination in the multicellular network (40, 41). Spontaneous widespread ICWs are rare to be seen in mature brains under physiological conditions; however, it can be observed in the communication of neural stem and progenitor cells to coordinate the proliferation and differentiation (42, 43).

#### 2.2.2 Cell Migration

Non-connecting tumor microtubes can also promote the invasion and proliferation of the glioma cells. Immunohistochemistry showed that the tumor microtubes were rich of actins, myosin IIa, and microtubules, and three-dimension scanning electron microscopy (3D SEM) showed that mitochondria and microvesicles traveled quickly and frequently in the tubes, implying active movement, ATP production, and vesicle trafficking (12, 44). It has been shown that the nucleus is able to travel in cellular membrane tubes after mitosis, which may be concerned with the invasion, treatment resistance, and cellular self-repairment and regeneration of glioma cells (12). Notably, it has been demonstrated that the synaptic and electrical integration described above also drives a calcium-dependent activation of glioma cell invasion (45) and promotes glioma proliferation (46).

According to the “seed and soil” hypothesis, it is not tumor cells that determine where to metastasize (47, 48). In contrast, the premetastatic niche is a prerequisite for the subsequent metastases of tumor cells (47, 48), supporting the fact that metastasis occurs only in selected organs but not in other organs, although tumor cells reach the vascular system of all organs (49). Residing in a specific niche is necessary for the survival and metastases of specific cells (50). The neurogenic niche in the subventricular zone (SVZ) of human lateral ventricles (51) is composed of neural stem cells (NSCs) and progenitor cells (NPCs), ependymal cells, astrocytes, microglia, macrophages, neurons, and extracellular matrix and associated vessels (52). The neurogenic niche is known to play an important role in the sustainment of the stem cell properties, including cell proliferation and self-renewal (53, 54). Similarly, in the perivascular niches in brain tumors, a cellular subpopulation of brain tumor stem–like cells (BTSCs) with NSC marker expression, including nestin and CD133, has also been identified (22). The perivascular niches were demonstrated to be related to the proliferation, self-renewal, invasion, and stemness of the tumor cells (55–57). Consequently, it is critical for tumor cells to be localized in the perivascular niches. It was seen that glioma stem–like cells extended TMs to move to the perivascular niche (12). A similar migration can be observed in the self-repairment of regeneration-damaged tumor cells (12). Likewise, it has been demonstrated that gliomas travel the same extracellular routes with migrant neural stem cells and neural progenitor cells during normal CNS development and damage repair (58–60). Above all, glioma cell migration induced by the growth of TMs is necessary for the invasion, malignancy progression, and recurrence of gliomas.

Normal neurodevelopment depends on the regulation of intracellular mechanisms, interactions with the microenvironment, and signaling pathways (22). Studies on TMs support the hypothesis that glioma progression may be seen as neurodevelopment with the loss of regulation and improperly reactivated, exploiting developmental pathways and molecules for TM formation and cell invasion (14, 22). The growth of TMs and the formation of a TM-connected network just like neurodevelopment are improperly reactivated at later time points of life.

#### 2.2.3 “Dendritic” Function in Electrical and Synaptic Integration of Glioma Into Neural Circuits

The newly discovered glutamatergic synaptic input observed in mouse models and resected patient tumor material with glutamate receptors of the AMPA subtype are usually located on tumor microtubes (45, 46), and activity-dependent, non-synaptic potassium currents (46) can activate intercellular calcium currents in the glioma cell network to drive a calcium-dependent activation of glioma cell invasion and promote glioma proliferation, which indicate that TMs may have a “dendritic” function for cancer cells. These findings are highly reminiscent of stem-cell populations regulated by a glutamatergic synaptic input, such as neuronal (61) and oligodendrocyte precursor cells (62) during normal neurodevelopment and function.

## 3 The Role of TMs in New Explanation for the Mechanisms of Resistance and Recurrence of Gliomas

It has been shown that TMs can mediate depolarization signals when subjected to stimulation such as radiotherapy and chemotherapy (12, 13). Apparently, even a slight fluctuation in intracellular calcium levels can cause great damage to intracellular homeostasis and impair cells and contribute to apoptotic cell death in glioma cells (63). It has been demonstrated that the synchronicity of the calcium peak of TM-connected cells is better than that of non-connected cells, implying that interconnecting tumor microtubes contribute to redistribute intracellular calcium to keep it at a nonlethal level and maintain intracellular homeostasis *via* membrane tubes connecting two cells and their forming networks to withstand adverse events (12). Intracellular calcium levels in cells without radiotherapy and TM-connected cells with radiotherapy were very homogeneous, while unconnected cells developed a high variability of calcium levels with radiotherapy (12). It has been shown that after radiotherapy, the vast majority of TM-connected glioma cells were protected from cell death, while most of the TM-unconnected and TM-negative cells died (12, 14). Moreover, glioblastoma cells may hijack neighboring nonmalignant astrocytes to transfer cGAMP *via* gap junctions as a result that activate the cGAS-STING pathway and release cytokines including IFNα and TNF to promote tumor metastasis (64) as it was demonstrated that melanoma cells can connect to active astrocytes *via* gap junctions to resist chemotherapy (65).

**Figure 2:**
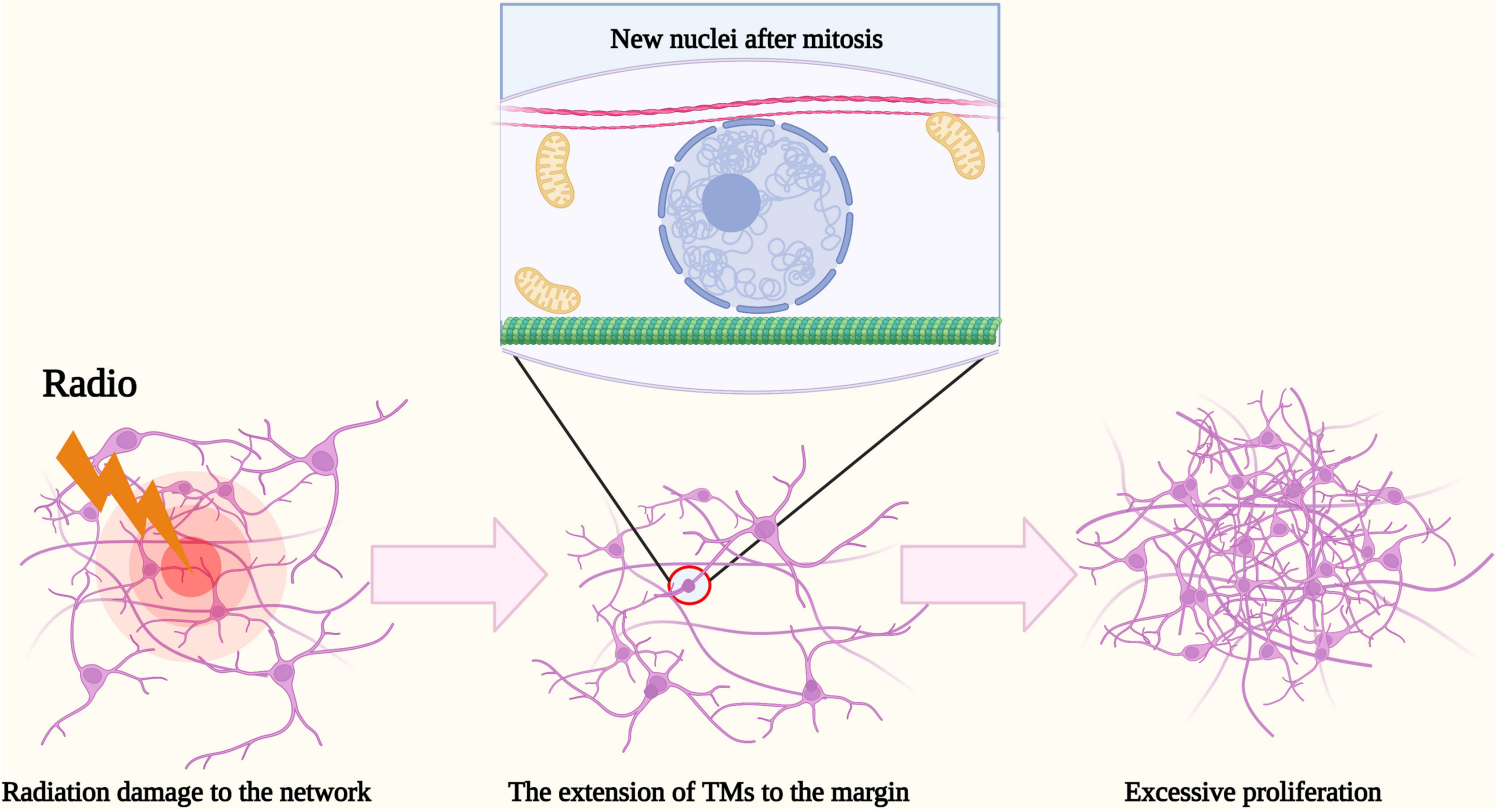
Schematic illustration of the excessive proliferation of glioma cells after radiation. After radiation damage to the network, the death of TM-connected tumor cell network resulted in a rapid extension of TMs of neighboring glioma cells into the margin. Within a few days, new nuclei were transmitted there through the TMs to facilitate the recurrence of glioma cells. The density of tumor cells in that region increased significantly and gradually even exceed those of unlesioned brain regions over time.

However, when the impairment is beyond their capacity of resistance, what would they do? It has been shown that the death of the TM-connected tumor cell network resulted in a rapid extension of the TMs of neighboring glioma cells into the lesion region, new TMs were extended toward the dead cells, and within a few days, new nuclei were transmitted through the tumor microtubes to the cells to facilitate the process of self-repairment (13). The density of tumor cells in that region increased significantly and gradually even exceed those of unlesioned brain regions over time to improve the resistance of damaged tumor cells and maintain the integrity of the tumor cell network (13) (**Figure 2**). In contrast, non-TM-connected glioma cells are infrequently expected to observe the self-repairing mechanism. It has been demonstrated that the number of TMs of astrogliomas is usually more than those of oligodendrocytes, while the length of the TMs of astrogliomas is also frequently longer than those of oligodendrocytes (12). Simultaneously, the number and the length of TMs appear to have a positive correlation of the grade and poor prognosis (12). According to the studies of Venkataramani et al. (45) and Venkatesh et al. (46), the electrical and synaptic integration of the TM-connected glioma cell network into neural circuits may dramatically promote glioma cell proliferation.

## 4 TMs Provides a New Angle to Understand the Incurability and Heterogeneity in Gliomas

Malignant gliomas are still regarded as a type of incurable tumor. Current clinical standard treatments for gliomas include radiotherapy, chemotherapy particularly with temozolomide (TMZ) (66, 67), and surgical resection (68). However, as a result of the widespread dissemination and robust recurrence of gliomas, the existing therapies are all limited.

After surgical resection, it has been shown that the vast majority of glioma cells tend to recur at or around the resection margin and even excessively proliferate (69), which means that, in fact, the resection of gliomas and the wound-healing response of a normal brain appear to promote the recurrence of gliomas. The phenomenon has also been observed in other tumors and has been attributed to the effect of wound-healing factors (70, 71) and growth factors (72). TMs may promote a new angle of explanation to the characteristic. S. Weil et al. proved that new TMs will be extended toward the margin of the resected region to promote the process of self-repairment after resection (13). Thus, more and more glioma cells recur, neighboring the margin and eventually exceeding the tumor cell density before the resected and that of the unresected region (13). Similarly, the similar self-repairment mechanism also applies to the reaction of gliomas after radiotherapy (12).

Additionally, although radiochemotherapy with temozolomide (TMZ) is still a mainstream therapy for gliomas, the poor diagnosis is attributed to the innate and acquired drug resistance of gliomas (68). O6-methylguanine-DNA methyltransferase (MGMT) protein, which is effective to repair DNA damage and consequently avoid cell apoptosis, is a major known mechanism for TMZ resistance (73). Clinical studies have shown that MGMT promoter hypermethylation in approximately half of gliomas appears to predict a better treatment response to TMZ (74). However, the nonresponders indicate that the novel mechanisms of the drug resistance should be explored further. It has also been demonstrated that gliomas with TMs connected are more resident to TMZ than those without TMs and also appear to excessively proliferate (13), which indicates an important role played in the TMZ resistance by the TM network. It may be attributed to the gap junction connections *via* Cx43 in glioma cells, which has been demonstrated to have a marked negative correlation of the TMZ resistance that may, in part, be attributed to mitochondrial apoptosis (75–77). Consequently, TM may be a potential therapeutic target for the TMZ resistance of gliomas. Recent research reveals that meclofenamate as a connexin43 blocker can impair the integrity of the TM network and increase the TMZ sensitivity of TMZ-resistant glioma cells (78).

Based on the discussion above, the number and length of TMs are highly influenced by the tumor type and grade, with a marked positive correlation of treatment resistance and poor prognosis (12). The explanation may be applied to the 1p/19q co-deleted gliomas.

Gliomas with 1p/19q co-deletion frequently predict a favorable diagnosis and a high responsiveness to various therapies, such as radiotherapy And chemotherapy, which is a characteristic for oligodendrogliomas but absent in astrocytomas (79). However, the potential molecular mechanisms remain indistinct. The function of the tumor microtubes and their forming networks may provide an explanation of this phenomenon. It has been demonstrated that TMs in 1p/19q non-co-deleted gliomas are frequently more and longer than those in 1p/19q co-deleted ones and the RNA-Seq gene expression data revealed that the gap junction protein Cx43 and core pathways driving neurite formation and the growth of neurite-like TMs are highly expressed in 1p/19q non-co-deleted gliomas (12). Since the genes of neurotrophic factors such as NGF and NT-4 (also called NTF4) that play a crucial role in the expression of GAP-43 and TTYH1 are demonstrated to be located on both chromosomal parts 1p and 19q, 1p/19q co-deleted gliomas may lack TMs due to downregulation of GAP-43 and their corresponding receptors (TrkA, TrkB), making them more susceptible to various therapies. As a consequence, the prognosis of oligodendroglioma is more favorable than that of astrocytoma (2, 66, 80–82).

## 5 TM-Connected Glioma Cells Share a Lot of Features With Glioma Stem-Like Cells

There is a view pointing out that the tumor development is a more abnormal organ development than tumor cell clones, implying that the principles of normal stem cell biology may also be applied to tumor development and cancer stem cells (83). The first purification of cancer stem-like cells is a leukemia-initiating cell isolated by Dick and his colleagues (84). Subsequently, a subpopulation of BTSCs was identified and also becomes a major area of interest within the field of neuro-oncology (5, 6). The BTSCs share a lot of features with normal neural stem cells (NSCs), such as they both have the ability of self-renewal and differentiation (22). CD133 (57), OCT4, NANOG and SOX2 (85) are all involved in well-established stemness markers in gliomas. However, it has been demonstrated that cells expressing different CSCs markers in gliomas are not distinguished by distinct functional properties or transcriptomic profiles; the difference is more likely to be a result of intrinsic tumor plasticity induced by the microenvironment (86). In recent years, there is growing evidence that BTSCs may play an important role in the treatment resistance and robust recurrence in gliomas (8, 9, 22). The studies of TMs may provide a new angle to understand and explain the associated mechanism in BTSCs.

According to the research of Xie and his colleagues in mouse models and resected patient material, TM-connected glioma cells share features with BTSCs (87). Compared with the TM-negative subpopulation, the TM-connected subpopulation performs significantly more genetic enrichment associated with both the embryonic stem cell status and the cell cycle (87). Nestin is known to be one of the best-established markers of cell stemness (57) and treatment resistance in gliomas (88). Compared with the TM-negative subpopulation, the TM-connected subpopulation performs a significantly higher expression of nestin and other stemness markers such as Musashi and Sox2 (87). Additionally, mice receiving the reimplantation of TM-connected glioma cells is shown to suffer obvious tumorigenesis with poor survival, while tumorigenesis fails to be detected in mice receiving the reimplantation of TM-negative glioma cells (87). The reimplanted TM-connected subpopulation is also demonstrated to reconstitute the heterogeneity of the TM content and network integration (87). Together, these findings suggest that TM-connected glioma cells share two typical features with BTSCs: proliferative potential and give rise to heterogeneous combinations of cells with different phenotypes (83).

Furthermore, after radiotherapy, it is shown that only nestin-positive tumor cells collectively extended more TMs and survive while nestin-negative tumor cells tend to fail to respond in such way and get impaired. Eventually, nestin-positive TM network–integrated glioma cells account for the vast majority of the tumor cells after radiotherapy. However, notably, both nestin-positive subpopulation with or without a TM response survived better, implying that the resistance mechanism induced by nestin is, in part, independent of that of TMs (87). According to the discussion above, the new findings may suggest a relationship between TM proficiency and cellular stemness.

It has been shown that reducing tumor bulk can induce the excessive proliferation of particularly quiescent BTSCs (89, 90). According to the discussion above, a similar mechanism has been detected in the self-repairment process induced by TMs (12, 13). Consequently, we speculate that responsive TMs might also be related to the tumor self-repairment induced by BTSCs for further research.

As a result that most standard therapies for glioma target proliferating cells, one of the crucial mechanisms of CSC treatment resistance is to switch into a quiescent state, particularly when the CSCs suffer from various standard therapies and a recent research showed that quiescent human glioma stem cells drove tumor initiation, infiltration, and recurrence following chemotherapy (91), which may be related to the dormancy of tumor cells in the perivascular niches (92). For example, it has been demonstrated that a cellular subpopulation of slow-cycling CSCs in GBM plays an important role in TMZ treatment resistance, and the resistance will be suppressed with the ablation of the slow-cycling CSCs (88). It is demonstrated that glioma cells may extend TMs to migrate to the specific perivascular niches with nuclei transport and contractile forces provided (12). It is the specific perivascular niche that determines the proliferating or quiescent state of tumor cells (47, 48). Consequently, we speculate that the dormancy process might be related to the rapid migration to the quiescent pool with TMs. Moreover, we speculate that other resistance mechanisms of TMs, such as ICW propagation, might also be applied to BTSC resistance for further research.

Although Xie and his colleagues have only demonstrated that TM-connected glioma cell networks are enriched for certain stem-like behaviors (87), it is highly likely that also invasive TM-positive but non-TM-connected glioma cells have stem-like properties for their many common features shared with neural progenitor and stem cells, which have been discussed above. Therefore, further research should be considered to unravel which stem/progenitor cell–like subpopulations are enriched in TM-positive cancer cells (unconnected or connected subpopulation) on the RNA expression level and on cellular functional levels.

## 6 Clinical Implications

Current clinical standard treatments for gliomas include radiotherapy, chemotherapy particularly with temozolomide (TMZ), and surgical resection. However, as a result of the robust resistance and self-repairment of gliomas, the existing therapies are all limited and there is an urgent need for a new treatment. TMs may provide a novel potential therapeutic target for gliomas.

### 6.1 Inhibition of Gap Junction

Since the gap junction is critical to the formation and communication of the network of tumor microtubes (12), we can inhibit the function of the gap junction to impair the integrity of the network. The available inhibitors may include 1) carbenoxolone (an effective medicine for gastric ulcer treatment but also shows a strong inhibitory effect on astrocytoma ICWs) (93), 2) inhibitors of ICW-propagating molecules (IP3, ATP receptors), 3) other calcium antagonists, such as mibefradil, and 4) inhibitors of various types of connexins. However, since various types of connexins, particularly connexin43, have crucial and complex functions to sustain intracellular homeostasis, the selection of the inhibitors must discreetly and considerably concentrate on the target specificity. The recent studies of Schneider and his colleagues in the human neocortical slice model showed a clear road for the introduction of TM network–targeting therapies into clinical concepts, proposing MFA as the first TM-targeted FDA-approved drug (78). They demonstrated that in comparison with TMZ treatment alone, TMZ and connexin43 blocker meclofenamate (MFA) co-treatment dramatically reduced interconnecting TMs and tumor bulk, achieving better therapeutic effect and prognosis (78). However, further research should be considered to evaluate the concentration levels of MFA within the human brain. In the environment, MFA monotherapy is being tested in patients with recurrent/progressive brain metastases from primary tumors in the United States (NCT02429570). In addition, based on the findings of Schneider et al. (78), a national phase I/II study of MFA/TMZ combination treatment in recurrent MGMT-methylated glioblastoma (“MecMeth” EudraCT2021-000708-39) is underway in Germany to measure the concentrations of MFA within gliomas, examine the safety and practicality of a combined MFA/TMZ strategy, and may acquire first insights into the effectiveness of MFA as the first clinically viable TM-targeted medication.

### 6.2 Blockade of Electrical and Synaptic Integration of TM-Connected Glioma Cells Into Neural Circuits

Glutamatergic synapses with the glutamate receptors of the AMPA subtype and activity-dependent potassium currents are crucial for glioma cell resistance, invasion, and proliferation (45, 46). The genetic and pharmacological blockade of AMPAR signaling may be applied to block the communication between neurons and glioma cells in order to inhibit the glioma progression and malignancy. According to clinical studies, epileptic seizures are common in individuals with gliomas (94), the recurrence or progression of which is linked to malignant glioma recurrence (95). The clinical manifestation was once thought to be due to the induction of glutamate released by brain tumors (96). However, a recent study suggested that excessive neural activity due to epilepsy might hasten glioma progression and malignancy (97), which is supported by the study of Venkataramani et al. (45). Together, these findings suggest that the clinically approved AMPAR-inhibiting antiepileptic medication perampanel (98) may be a new potential drug for gliomas. Lange et al. demonstrated anti-tumorigenic effects mediated by perampanel *in vitro* (99). In addition, the study of Salmaggi et al. has demonstrated that perampanel showed a pro-apoptotic effect on human glioma cell lines when used alone and also showed synergistic cooperativity when combined with TMZ (100). Against this backdrop, further preclinical studies and clinical trials should be considered.

### 6.3 Inhibition of the Growth of TMs

There are two known factors to be demonstrated to direct the growth of TMs: GAP-43 and Ttyh1 (12). Neurotrophic factors such as NGF and NT-4 (also called NTF4) promote the expression of GAP-43 (12). Silencing genes and disrupting signaling pathways related to the expression of GAP-43 and Ttyh1 may be potential choices.

### 6.4 Regulation of Associated Signaling Pathway in Tumor Microenvironment

Recent research showed that the downregulation of NOTCH1 is effective to promote the growth of TMs (101). However, since it was also demonstrated to play an important role in the perivascular niche of resistance in gliomas (101), additional potential signaling pathways or specific blockers remain to be identified. Gritsenko et al. recently demonstrated that intercellular adhesion and signaling provided by p120-catenin–dependent adherens junctions is indispensable for TM-connected glioma cell progression and malignancy, implying that p120-catenin–dependent adherens junctions or their downstream effectors may be a potential target (36).

### 6.5 Hijack the Network for Drug Transport

It is a potential way of therapy to hijack the network to distribute injected toxic molecules, which are gap junction permeable (24). In addition, it has been demonstrated that lipopolysaccharide-anchored macrophages can hijack tumor microtube networks for selective drug transport, serving as versatile bioactive carriers of drugs such as Dox and repressing tumor genesis (102).

## 7 Conclusion and Perspectives

The newly discovered TMs and their forming network may provide a new angle to understand the resistance and recurrence of incurable gliomas. TMs share a lot of features in development and function with the axons of immature neural cells and may be seen as “electric synapse” connecting glioma cells and mediating intercellular communication. Both TM-connected cells and immature neural cells can be integrated into a multicellular network and enrich cell stemness (22, 87).

The findings may support the hypothesis that gliomas are initiated by cancer stem cells (5, 7, 57, 103). In essence, glioma progression can be seen as neurodevelopment improperly reactivated at later time points of life (22). The subpopulation of cancer stem cells has been demonstrated to play a crucial role in the robust resistance and recurrence in gliomas (7, 104). Recent research showed that glioma cells with stemness feature tend to extend TMs and integrate into a network to withstand adverse events (87), which might provide a new angle to understand the resistance and recurrence mechanism of glioma stem–like cells. However, further research should be undertaken to investigate the stem cell behavior of TM-connected nestin-positive glioma cells and the existence of the subpopulation of TM connected with the expression of other stemness markers. In previous studies, BTSCs and TM-connected glioma cells are always discussed as two independent subpopulations. In this review, we discuss the overlap between them and appeal to do a further comprehensive study. TMs connect glioma cell morphology with the molecular phenotype, suggesting network integration as a new potential signature of cancer stem cells. A similar mechanism may be also applied to other tumors with a membrane tube connection such as breast cancer (105, 106), cervix cancer (107), leukemia (108–110), and lung cancer (111), which require further research. Overall, TM-connected cells provide a novel potential therapeutic target subpopulation for gliomas and might also be a target of resistant cancer stem cells after further research.

## Author Contributions

The work presented here was carried out in collaboration among all authors. HS conceived this work. XW designed the review and drafted the manuscript. HS revised the manuscript. All authors read, commented on, and approved this manuscript.

## Funding

Guangdong Basic and Applied Basic Research Foundation (2020A1515010038); the Presidential Foundation of Zhujiang Hospital of Southern Medical University (No. yzjj2018rc03); University students innovation and entrepreneurship project “Three-dimensional visualization of glioma vessels based on tissue clearing technique” (No.202112121004)

## Conflict of Interest

The authors declare that the research was conducted in the absence of any commercial or financial relationships that could be construed as a potential conflict of interest.

## Publisher’s Note

All claims expressed in this article are solely those of the authors and do not necessarily represent those of their affiliated organizations, or those of the publisher, the editors and the reviewers. Any product that may be evaluated in this article, or claim that may be made by its manufacturer, is not guaranteed or endorsed by the publisher.

## Abbreviations

TM, tumor microtube; NSC, neural stem cell; CNS, central nervous system; Gap43, growth-associated protein 43; Cx43, connexin 43; ICWs, intercellular calcium waves; SVZ, subventricular zone; NPCs, neural progenitor cells; BTSCs, brain tumor stem-like cells; TMZ, temozolomide.
